# Enhancing early mortality prediction for sepsis-associated acute respiratory distress syndrome patients via optimized machine learning algorithm: development and multiple databases’ validation of the SAFE-Mo

**DOI:** 10.1097/JS9.0000000000002741

**Published:** 2025-06-25

**Authors:** Luofeng Jiang, Chuting Yu, Chaoran Xie, Yongjun Zheng, Zhaofan Xia

**Affiliations:** a Department of Burn Surgery, The First Affiliated Hospital of Naval Medical University, Shanghai, China; b Research Unit of Key Techniques for Treatment of Burns and Combined Burns and Trauma Injury, Chinese Academy of Medical Sciences, Shanghai, China; c Department of Digestive, The First Affiliated Hospital of Naval Medical University, Shanghai, China

**Keywords:** acute respiratory distress syndrome, eICU CRD, machine learning, MIMIC-IV, NWICU, sepsis, sepsis-induced acute lung injury

## Abstract

**Background:**

Acute respiratory distress syndrome (ARDS) is associated with high mortality, with sepsis accounts for 31–34% of cases. Given the global burden of sepsis (508 cases per 100 000 person-years) and its association with 20% of all global deaths, early mortality prediction in patients with sepsis-associated ARDS is critical. This study developed and validated the Sepsis-associated ARDS Fatality Evaluation Model (SAFE-Mo), a machine learning (ML) model designed to predict early mortality in sepsis-associated ARDS patients, enabling earlier identification of high-risk individuals.

**Methods:**

Data were extracted from the Medical Information Mart for Intensive Care IV (MIMIC-IV, v3.0), eICU Collaborative Research Database (eICU CRD, v2.0), and Northwest ICU (NWICU, v0.1.0) using Structured Query Language (SQL). SAFE-Mo was constructed using ML algorithm (svmRadialSigma) focusing on median survival days among deceased patients as the primary outcome. The model’s performance was validated externally using the MIMIC-IV and eICU CRD database and compared against four commonly used clinical risk assessment models (acute physiology score III (APSIII), simplified acute physiology score II (SAPS II), sequential organ failure assessment (SOFA), Charlson comorbidity index (CCI)). Additionally, NWICU was used to further validate SAFE-Mo’s generalization. Discrimination, calibration, and clinical utility were evaluated using area under the curve (AUC), Decision Curve Analysis (DCA), and calibration curves.

**Results:**

SAFE-Mo demonstrated superior predictive capability of early mortality compared to traditional models. It showed the largest reasonable risk threshold probability range and highest net benefit. Calibration curves indicated a slight overestimation of mortality risk overall. With our simple SAFE-Mo web page, SAFE-Mo can assist clinicians in identifying high-risk patients early, like patients with unusually high levels of lactate in sepsis-associated ARDS, assessing prognosis, and facilitating risk-adjusted comparisons of center-specific outcomes. Practical advantages include guiding personalized treatment strategies, determining the need for aggressive interventions, and optimizing resource utilization.

**Conclusion:**

This study utilized the MIMIC-IV, eICU CRD, and NWICU databases to construct and validate a ML model, SAFE-Mo, which predicts early mortality in patients with sepsis-associated ARDS and outperforms traditional prediction models across all metrics. SAFE-Mo can guide clinicians to focus on critical indicators such as lactate, urine output, anion gap, and others, enabling appropriate measures to improve clinical outcomes for high-risk patients.

## Introduction

Acute respiratory distress syndrome (ARDS) is an acute, life-threatening condition characterized by noncardiogenic pulmonary edema, severe hypoxemia, and decreased lung compliance. Its global annual incidence is estimated at 1.5–75 cases per 100 000 person-years, with mortality rates as high as 46.1%^[1,2]^. Sepsis is an uncontrolled inflammatory response to infection. According to the Global Burden of Disease Study analysis for 1990–2017, the age-standardized incidence of sepsis in 2017 was 508.4 per 100 000 person-years, and at least 20% of all deaths were sepsis-related^[3]^. Sepsis is one of the most common causes of ARDS, with approximately 31.4–33.8% of sepsis patients developing ARDS^[4,5]^. The coexistence of sepsis and ARDS markedly worsens patient outcomes^[4-6]^. Notably, most patients with sepsis-associated ARDS face significant challenges, characterized by a high short-term mortality rate that remains a pressing concern in clinical practice^[1,7]^. These intertwined epidemiological features underscore the need for early risk identification in patients with sepsis-associated ARDS. Accurate prediction of patient outcomes—thereby facilitating timely adjustments in treatment paradigms—has become an urgent priority for clinicians.

Recent advancements in machine learning (ML) have garnered considerable attention within the medical community^[8]^. Compared to traditional logistic regression or Cox proportional hazards models, ML techniques exhibit superior performance in disease prediction and classification^[9,10]^. However, existing studies on ML applications for predicting outcomes in sepsis-associated ARDS still present opportunities for improvement^[11–13]^. Currently, these studies exhibit one or more of the following specific limitations: (1) reliance on outdated database versions; (2) validation on a single cohort with a limited sample size; (3) lack of publicly available source code, compromising transparency; (4) suboptimal area under the curve (AUC) performance; (5) absence of clinical validation, precluding immediate application. These shortcomings underscore the imperative for further research aimed at improving predictive accuracy and enhancing the overall reliability and robustness of the findings.

To address these challenges, a ML -based prediction model was developed, focusing on median survival days among deceased patients as the primary outcome. The objective was to identify characteristics associated with short-term mortality in patients with sepsis-associated ARDS, thereby enabling earlier identification and potentially reducing short-term mortality rates. In pursuit of this goal, multiple ML algorithms and multiple independent databases were employed to construct and validate the sepsis-associated ARDS fatality evaluation model (SAFE-Mo). It is anticipated that SAFE-Mo will not only predict short-term mortality effectively but also contribute to the development of more effective assessment criteria for subsequent clinical management. Besides, our study is compliant with the TITAN Guidelines 2025—governing declaration and use of AI^[14]^.

The establishment of SAFE-Mo represents a step towards improving the prognosis and quality of life for patients suffering from sepsis-associated ARDS. Through precise predictions and early interventions, this study aims to provide a robust foundation for enhancing clinical decision-making processes and ultimately improving patient outcomes.

## Methods

### Data source

All the clinical data of the patients with sepsis in this study were obtained from the source: the MIMIC-IV (https://physionet.org) v3.0^[15]^ database, the eICU (CRD, https://physionet.org) v2.0^[16,17]^, and the NWICU v0.1.0^[18]^. One author completed the Collaborative Institutional Training Initiative (CITI) Program (No.50140789), obtained access to the MIMIC-IV, eICU CRD, and NWICU databases, and signed the data use agreement (Supplemental Digital Content, Table S1 http://links.lww.com/JS9/E462).

### Participants

Patients diagnosed with sepsis according to the International Classification of Diseases, 9th Revision (ICD-9: 995.91, 995.92, 785.52), and meeting the diagnostic criteria for Acute Respiratory Distress Syndrome (ARDS)^[19]^ were included in the study. Detailed inclusion criteria for each database are provided in Supplemental Digital Content, Table S2 http://links.lww.com/JS9/E463. Patients with missing values >20% or patients younger than 18 years of age or those who did not develop ARDS following the diagnosis of sepsis were excluded from the study. Given the hypothesis-driven epidemiological nature of the study, all patients eligible within the MIMIC-IV, eICU CRD, and NWICU database were included to maximize statistical power. Thus, a sample size calculation was not performed for this investigation.

### Variable extraction

The following information of the patients from the MIMIC-IV, eICU CRD, and NWICU databases was used: (1) demographic characteristics (age, sex, admission type, insurance, marital status, race and degree), (2) vital signs (heart rate, respiratory rate, body temperature, systolic and diastolic blood pressure, mean blood pressure), (3) clinical risk assessment models (SOFA^[20]^, APS III^[21]^, SAPS II^[22]^ and CCI^[23]^). (4) Laboratory tests (SpO_2_, glucose, urine output, lactate, pH, SO_2_, PO_2_, PCO_2_, Arterial-Alveolar Oxygen Difference Calculation (A-aDO_2_-calc), Base excess, total CO_2_, calcium, potassium, sodium, platelets, WBC, albumin, anion gap, blood urea nitrogen (BUN), creatinine, abs monocytes, abs neutrophils, international normalized ratio (INR), prothrombin time (Pt), partial thromboplastin time (Ptt), alanine aminotransferase (ALT), alkaline phosphatase (ALP), aspartate aminotransferase (AST), and total bilirubin). (5) Comorbidities (myocardial infarction, congestive heart failure, peripheral vascular disease, cerebrovascular disease, dementia, chronic pulmonary disease, rheumatic disease, peptic ulcer disease, mild liver disease, diabetes without chronic complication (CC), diabetes with CC, paraplegia, renal disease, malignant cancer, severe liver disease, and metastatic solid tumor).

### Data preprocessing

For laboratory tests, data from within 24 hours prior to patient admission were included. Data points for each variable were selected based on the maximum and minimum values present within the datasets. In the context of disease scoring systems, only the initial test values utilized for analysis were included. To minimize bias due to missing data while preserving data integrity and ensuring robust statistical analyses, we first excluded variables with >40% missing values. Subsequently, patients with >20% missing data in the final cohort were excluded. Missing data were addressed using multiple imputation (MI) with the mice package^[24]^ in R. To simulate potential clinical scenarios, we retained the 100% missing model variables in the NWICU dataset. Those variables (urine output, A-aDO_2_-calc max, A-aDO_2_-calc min, and potassium min) in NWICU were replaced with the median from MIMIC-IV training set. For the detection of imputation results, we employed several methods: the default method (meth), Bayesian linear regression (norm), bootstrap-based linear regression (norm.boot), linear regression prediction (norm.predict), classification and regression trees (CART), and random forests (rf). The optimal MI algorithm repeated 50 times and the average of the results was used to impute the missing data for subsequent analysis.

### Feature selection

Multicollinearity was assessed using the Kappa statistic, with a kappa value of ≤ 100 indicating minimal multicollinearity, 100 < kappa ≤ 1000 suggesting moderate multicollinearity, and kappa > 1000 indicating severe multicollinearity. To mitigate the impact of multicollinearity among multiple variables on model construction (Supplemental Digital Content, Fig. S2 http://links.lww.com/JS9/E461), we employed Lasso regression for data processing. Ten-fold cross-validation was employed to identify robustness of Lasso and acquire the minimum lambda value to select features for the following model establishment section.

### Model establishment

To reflect the mean level of patient mortality risk, we used the median of survival time as a cutoff for model construction (Supplemental Digital Content, Fig. S1, http://links.lww.com/JS9/E461). The MIMIC-IV was randomly split into training (80%) set and internal validation (20%) set. Stratified sampling (compared with random sampling in Supplemental Digital Content, Table S3 http://links.lww.com/JS9/E464) was performed using R package createDataPartition to ensure balanced class distribution in both training and validation sets for post hoc model evaluation. A total of 36 ML algorithms were employed to build the models, with AUC serving as the evaluation metric. Ten-fold cross-validation was used to assess model stability. These algorithms included Generalized Linear Models (vglmContRatio, vglmCumulative, bayesglm, glmboost, glm), XGBoost (xgbLinear, xgbTree), Regularization Methods (glmnet), Probabilistic Discriminant Analysis (pda, pda2), Random Forest (rf, RRF, cforest), Decision Trees (C5.0, C5.0Tree), Kernel Methods (kernelpls, pls, simpls, widekernelpls, spls), Neural Networks (nnet, pcaNNet), Recursive Partitioning and Regression Trees (rpart), Conditional Inference Trees (ctree), Support Vector Machines (svmLinear, svmRadial, svmRadialCost, svmRadialSigma), and other ML algorithms (LogitBoost, fda, kknn, knn, lda, lda2, earth, gcvEarth). The optimal model was selected as the SAFE-Mo according to the best AUC in the internal validation set. The primary outcome measure of this study was the prediction of 26-day mortality in patients with sepsis-associated ARDS.

### Model validation and comparison

To improve the credibility of SAFE-Mo, both internal and external validations were performed. The internal validation set was from MIMIC-IV database. External validation was carried out using two independent datasets: one from the eICU-CRD database and the other from the NWICU database. To evaluate the performance of SAFE-Mo, in internal validation set, we first conducted a systematic assessment of all essential binary classification metrics (accuracy, precision, recall, F1-score, specificity, sensitivity, negative predictive value (NPV), and positive predictive value (PPV)) through bootstrap resampling (R = 100 iterations) to ensure robust estimation. Subsequently, Wilcoxon analysis, univariate analysis, and multivariate logistic regression were utilized to contrast it with four commonly used clinical risk assessment models (APS III, SAPS II, SOFA, CCI) in external validation set one. External validation set two was used as an independent dataset that more closely reflected real-world clinical scenarios to further validate the clinical applicability of SAFE-Mo. Additionally, Pearson correlation analysis was performed to compare the correlation between SAFE-Mo and the four clinical risk assessment models. A logistic model was established, combining the optimal model with the four clinical risk assessment models.

Furthermore, the performance of SAFE-Mo and previous clinical risk assessment models were evaluated using three dimensions: discrimination, calibration, and clinical utility. Discrimination was quantitatively assessed using the AUC. Decision Curve Analysis (DCA) curves were drawn using the rmda package^[25]^ in R to further evaluate its clinical application value. Calibration was evaluated through the consistency of predicted probabilities and graphical representations of observed outcomes based on 1000 bootstrap resamples.

### Statistical analysis

(1) Data extraction and preprocessing platforms. Data from the MIMIC-IV, eICU CRD, and NWICU databases were extracted and integrated using the SQL. SQL scripts were implemented using ProstgreSQL (v17.2, https://www.postgresql.org/) and Navicat Premium 16 (Navicat, v16.3.11, https://www.navicat.com.cn/products/navicat-premium). (2) Methods for variable description. Continuous variables were presented as mean ± standard deviation (mean ± SD) if they satisfied the normality assumption by the Shapiro–Wilk test; otherwise, they were reported as median (interquartile range). Categorical variables were expressed as counts or percentages. (3) Statistical methods for group comparisons. For continuous variables, Student’s *t*-test was used if the normality assumption was met; otherwise, the Wilcoxon rank-sum test was applied. Categorical variables were analyzed using Pearson’s chi-squared (*χ*^2^) test, with Fisher’s exact test employed when any expected cell count was <5. A two-sided *P*-value < 0.05 was considered statistically significant. (4) Statistical software. Statistical analysis and data visualization was performed using R software (Version 4.3.3, The R Project for Statistical Computing, https://www.r-project.org/). All selections of statistical tests and statistical calculations were performed using the tableone package. All graphics were created with the ggplot2 package.

## Results

### Baseline data characteristics

**Figure 1.**
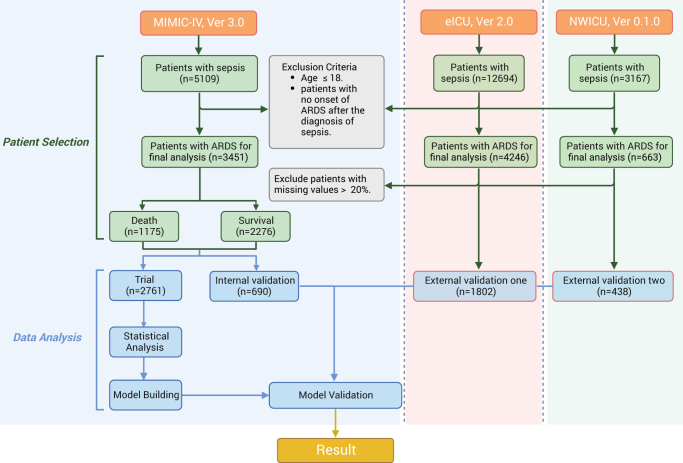
Workflow diagram. *MIMIC-IV* Medical Information Mart for Intensive Care IV, *eICU-CRD* eICU Collaborative Research Database. *NWICU* Northwest ICU.

According to the workflow depicted in Figure 1, a total of 3451 patients from the MIMIC-IV database were included in this study. Among these participants, 2276 survived, while 1175 did not survive. The median survival time for the deceased cohort was 26 days (Supplemental Digital Content, Fig. S1, http://links.lww.com/JS9/E461). Baseline characteristics were analyzed with a focus on 26-day mortality as the primary outcome (Table 1).

**Table 1:** Baseline table

Variables	Death	Survival	*P* value
Number (sample size)	1175	2276	
Demographic characteristics			
Gender (*n*, mean ± SD)			0.15
Male	635 ± 54.0	1290 ± 56.7	
Female	540 ± 46.0	986 ± 43.3	
Admission type (*n*, mean ± SD)			<0.001
Emergency	849 ± 72.3	1683 ± 73.9	
Surgery	9 ± 0.8	65 ± 2.9	
Urgent	305 ± 26.0	502 ± 22.1	
Elective	8 ± 0.7	24 ± 1.1	
Observation	4 ± 0.3	2 ± 0.1	
Insurance (*n*, mean ± SD)			0.027
Medicare	773 ± 65.8	1378 ± 60.5	
Private	242 ± 20.6	545 ± 23.9	
Medicaid	139 ± 11.8	310 ± 13.6	
Other	21 ± 1.8	43 ± 1.9	
Marital status (*n*, mean ± SD)			0.003
Married	529 ± 45.0	1056 ± 46.4	
Single	341 ± 29.0	744 ± 32.7	
Divorced	110 ± 9.4	194 ± 8.5	
Widowed	195 ± 16.6	282 ± 12.4	
Race (*n*, mean ± SD)			0.144
White	822 ± 70.0	1652 ± 72.6	
Black	158 ± 13.4	299 ± 13.1	
Asian	66 ± 5.6	92 ± 4.0	
Other	129 ± 11.0	233 ± 10.2	
Vital signs			
Age (year, mean ± SD)	70.99 ± 14.37	67.04 ± 15.56	<0.001
Heart rate min (times/minute, mean ± SD)	76.60 ± 17.60	77.20 ± 15.97	0.312
Heart rate max (times/minute, mean ± SD)	114.30 ± 22.44	112.43 ± 21.02	0.015
SBP min (mmHg, mean ± SD)	77.47 ± 14.41	84.66 ± 12.96	<0.001
SBP max (mmHg, mean ± SD)	139.55 ± 20.65	144.55 ± 20.38	<0.001
DBP min (mmHg, mean ± SD)	38.64 ± 10.08	42.59 ± 9.30	<0.001
DBP max (mmHg, mean ± SD)	84.04 ± 18.29	86.01 ± 17.65	0.002
MBP min (mmHg, mean ± SD)	49.10 ± 11.35	54.37 ± 9.99	<0.001
MBP max (mmHg, mean ± SD)	97.95 ± 17.80	100.56 ± 17.20	<0.001
Respiratory rate min (times/minute, mean ± SD)	13.57 ± 4.20	13.35 ± 3.81	0.115
Respiratory rate max (times/minute, mean ± SD)	30.58 ± 6.04	29.65 ± 6.13	<0.001
Temperature min (°C, mean ± SD)	36.16 ± 0.67	36.37 ± 0.59	<0.001
Temperature max (°C, mean ± SD)	37.44 ± 0.91	37.76 ± 0.90	<0.001
Laboratory tests			
SPO_2_ min (%, mean ± SD)	90.65 ± 4.44	91.93 ± 4.02	<0.001
Urine output (mL, mean ± SD)	1262.56 ± 1313.86	2023.21 ± 1411.44	<0.001
Lactate min (mmol/l, mean ± SD)	2.20 ± 1.01	1.57 ± 0.80	<0.001
Lactate max (mmol/l, mean ± SD)	4.04 ± 2.46	2.55 ± 1.89	<0.001
pH min (mean ± SD)	7.24 ± 0.14	7.30 ± 0.11	<0.001
pH max (mean ± SD)	7.38 ± 0.09	7.40 ± 0.07	<0.001
PO_2_ min (mmHg, mean ± SD)	61.37 ± 27.67	69.62 ± 29.71	<0.001
PO_2_ max (mmHg, mean ± SD)	179.39 ± 109.91	177.59 ± 109.01	0.647
PCO_2_ min (mmHg, mean ± SD)	34.56 ± 9.04	35.68 ± 8.19	<0.001
PCO_2_ max (mmHg, mean ± SD)	48.85 ± 13.80	47.61 ± 13.17	0.01
A-aDO_2_-Calc min (mean ± SD)	217.24 ± 115.99	181.24 ± 103.59	<0.001
A-aDO_2_-Calc max (mean ± SD)	404.64 ± 172.19	337.91 ± 170.48	<0.001
Base excess min (mean ± SD)	−7.82 ± 7.44	−4.35 ± 5.85	<0.001
Base excess max (mean ± SD)	−2.21 ± 5.26	−0.49 ± 4.24	<0.001
Total CO_2_ min (mmHg, mean ± SD)	19.23 ± 6.57	21.77 ± 5.72	<0.001
Total CO_2_ max (mmHg, mean ± SD)	24.25 ± 5.98	25.66 ± 5.18	<0.001
Platelets min (1 × 10^9^/L, mean ± SD)	166.61 ± 109.45	191.60 ± 106.60	<0.001
Platelets max (1 × 10^9^/L, mean ± SD)	214.02 ± 126.27	236.69 ± 123.67	<0.001
WBC min (1 × 10^9^/L, mean ± SD)	11.90 ± 7.14	11.18 ± 6.25	0.002
WBC max (1 × 10^9^/L, mean ± SD)	16.50 ± 8.92	15.72 ± 8.11	0.01
Anion gap min (mmol/l, mean ± SD)	15.09 ± 3.89	13.16 ± 3.20	<0.001
Anion gap max (mmol/l, mean ± SD)	19.58 ± 4.95	17.16 ± 4.35	<0.001
BUN min (mg/dL, mean ± SD)	36.02 ± 19.73	27.84 ± 17.95	<0.001
BUN max (mg/dL, mean ± SD)	42.20 ± 21.94	33.76 ± 20.81	<0.001
Creatinine min (mg/dL, mean ± SD)	1.70 ± 0.97	1.35 ± 0.86	<0.001
Creatinine max (mg/dL, mean ± SD)	2.10 ± 1.15	1.69 ± 1.05	<0.001
Abs basophils min (1 × 10^9^/L, mean ± SD)	0.01 ± 0.02	0.02 ± 0.02	0.069
Abs basophils max (1 × 10^9^/L, mean ± SD)	0.02 ± 0.03	0.02 ± 0.03	0.002
Abs eosinophils min (1 × 10^9^/L, mean ± SD)	0.03 ± 0.05	0.04 ± 0.05	0.034
Abs eosinophils max (1 × 10^9^/L, mean ± SD)	0.05 ± 0.07	0.06 ± 0.07	0.008
Abs lymphocytes min (1 × 10^9^/L, mean ± SD)	0.88 ± 0.60	0.88 ± 0.58	0.922
Abs lymphocytes max (1 × 10^9^/L, mean ± SD)	1.05 ± 0.67	1.06 ± 0.64	0.678
Abs monocytes min (1 × 10^9^/L, mean ± SD)	0.49 ± 0.35	0.48 ± 0.33	0.446
Abs monocytes max (1 × 10^9^/L, mean ± SD)	0.60 ± 0.41	0.60 ± 0.38	0.677
Abs neutrophils min (1 × 10^9^/L, mean ± SD)	11.01 ± 7.08	10.51 ± 6.25	0.032
Abs neutrophils max (1 × 10^9^/L, mean ± SD)	12.26 ± 7.64	12.07 ± 7.05	0.471
INR min (mean ± SD)	1.48 ± 0.37	1.36 ± 0.30	<0.001
INR max (mean ± SD)	1.83 ± 0.65	1.55 ± 0.50	<0.001
Pt min (seconds, mean ± SD)	16.17 ± 3.82	14.96 ± 3.10	<0.001
Pt max (seconds, mean ± SD)	19.47 ± 6.20	16.78 ± 4.80	<0.001
Ptt min (seconds, mean ± SD)	35.40 ± 8.37	32.40 ± 7.00	<0.001
Ptt max (seconds, mean ± SD)	44.99 ± 15.65	38.56 ± 12.97	<0.001
ALT min (mean ± SD)	39.65 ± 31.07	37.03 ± 28.83	0.014
ALT max (mean ± SD)	48.99 ± 39.77	43.55 ± 36.26	<0.001
ALP min (mean ± SD)	102.19 ± 52.55	98.47 ± 50.82	0.044
ALP max (mean ± SD)	115.87 ± 60.24	108.10 ± 57.93	<0.001
AST min (mean ± SD)	65.58 ± 46.25	52.27 ± 41.15	<0.001
AST max (mean ± SD)	84.99 ± 65.89	64.98 ± 57.39	<0.001
Total bilirubin min (mg/dL, mean ± SD)	1.14 ± 0.92	0.94 ± 0.84	<0.001
Total bilirubin max (mg/dL, mean ± SD)	1.42 ± 1.19	1.15 ± 1.08	<0.001
Hematocrit min (%, mean ± SD)	29.15 ± 5.99	29.40 ± 5.74	0.228
Hematocrit max (%, mean ± SD)	34.08 ± 6.11	33.78 ± 5.96	0.172
Bicarbonate min (mmol/L, mean ± SD)	18.14 ± 5.85	20.53 ± 5.05	<0.001
Bicarbonate max (mmol/L, mean ± SD)	22.18 ± 5.21	23.92 ± 4.46	<0.001
Glucose min (mg/dL, mean ± SD)	107.78 ± 37.67	112.29 ± 32.01	<0.001
Glucose max (mg/dL, mean ± SD)	171.57 ± 66.59	165.74 ± 60.42	0.01
Hemoglobin min (g/dL, mean ± SD)	9.44 ± 1.96	9.67 ± 1.93	0.001
Hemoglobin max (g/dL, mean ± SD)	10.98 ± 2.02	11.04 ± 2.01	0.387
Calcium min (mmol/L, mean ± SD)	7.60 ± 0.88	7.67 ± 0.79	0.027
Calcium max (mmol/L, mean ± SD)	8.35 ± 0.82	8.30 ± 0.73	0.053
Potassium min (mmol/L, mean ± SD)	3.98 ± 0.67	3.80 ± 0.60	<0.001
Potassium max (mmol/L, mean ± SD)	4.75 ± 0.76	4.52 ± 0.73	<0.001
Sodium min (mmol/L, mean ± SD)	136.06 ± 5.70	136.23 ± 4.83	0.338
Sodium max (mmol/L, mean ± SD)	139.61 ± 5.67	139.47 ± 4.66	0.456
Comorbidities			
Myocardial infarct (*n*, mean ± SD)			0.002
No	959 ± 81.6	1952 ± 85.8	
Yes	216 ± 18.4	324 ± 14.2	
Congestive heart failure (*n*, mean ± SD)			0.112
No	739 ± 62.9	1495 ± 65.7	
Yes	436 ± 37.1	781 ± 34.3	
Peripheral vascular disease (*n*, mean ± SD)			0.056
No	1013 ± 86.2	2015 ± 88.5	
Yes	162 ± 13.8	261 ± 11.5	
Cerebrovascular disease (*n*, mean ± SD)			0.277
No	1036 ± 88.2	2036 ± 89.5	
Yes	139 ± 11.8	240 ± 10.5	
Dementia (*n*, mean ± SD)			0.086
No	1137 ± 96.8	2226 ± 97.8	
Yes	38 ± 3.2	50 ± 2.2	
Chronic pulmonary disease (*n*, mean ± SD)			0.283
No	795 ± 67.7	1582 ± 69.5	
Yes	380 ± 32.3	694 ± 30.5	
Rheumatic disease (*n*, mean ± SD)			0.499
No	1121 ± 95.4	2184 ± 96.0	
Yes	54 ± 4.6	92 ± 4.0	
Peptic ulcer disease (*n*, mean ± SD)			0.843
No	1137 ± 96.8	2198 ± 96.6	
Yes	38 ± 3.2	78 ± 3.4	
Mild liver disease (*n*, mean ± SD)			<0.001
No	782 ± 66.6	1818 ± 79.9	
Yes	393 ± 33.4	458 ± 20.1	
Diabetes without CC (*n*, mean ± SD)			0.677
No	851 ± 72.4	1665 ± 73.2	
Yes	324 ± 27.6	611 ± 26.8	
Diabetes with CC (*n*, mean ± SD)			0.104
No	1089 ± 92.7	2071 ± 91.0	
Yes	86 ± 7.3	205 ± 9.0	
Paraplegia (*n*, mean ± SD)			0.04
No	1140 ± 97.0	2174 ± 95.5	
Yes	35 ± 3.0	102 ± 4.5	
Renal disease (*n*, mean ± SD)			0.009
No	830 ± 70.6	1703 ± 74.8	
Yes	345 ± 29.4	573 ± 25.2	
Malignant cancer (*n*, mean ± SD)			<0.001
No	927 ± 78.9	1947 ± 85.5	
Yes	248 ± 21.1	329 ± 14.5	
Severe liver disease (*n*, mean ± SD)			<0.001
No	1004 ± 85.4	2121 ± 93.2	
Yes	171 ± 14.6	155 ± 6.8	
Metastatic solid tumor (*n*, mean ± SD)			<0.001
No	1011 ± 86.0	2155 ± 94.7	
Yes	164 ± 14.0	121 ± 5.3	
AIDS (*n*, mean ± SD)			0.003
No	1172 ± 99.7	2245 ± 98.6	
Yes	3 ± 0.3	31 ± 1.4	
Clinical risk assessment models			
APSIII (mean ± SD)	78.76 ± 26.89	58.20 ± 22.28	<0.001
SAPSII (mean ± SD)	56.47 ± 16.26	42.90 ± 14.64	<0.001
SOFA-24 hours (mean ± SD)	2.86 (2.87)	1.38 (1.58)	<0.001
CCI (mean ± SD)	6.61 ± 2.79	5.14 ± 2.82	<0.001

A-aDO_2_—calc, Arterial-Alveolar Oxygen Difference Calculation; Abs, Absolute; AIDS, Acquired Immunodeficiency Syndrome; ALP, Alkaline Phosphatase; ALT, Alanine Aminotransferase; AST, Aspartate Aminotransferase; APSIII, Acute Physiology Score III; BUN, Blood Urea Nitrogen; CC, Chronic Complication; CCI, Charlson Comorbidity Index; DBP, Diastolic Blood Pressure; INR, International Normalized Ratio; max, Maximum, MBP, Mean Blood Pressure; min, Minimum; n, number; Pt, Prothrombin Time; Ptt, Partial Thromboplastin Time; SAPSII, Simplified Acute Physiology Score II; SBP, Systolic Blood Pressure; SD, Standard Deviation; SOFA, sequential organ failure assessment; SpO_2_, Oxygen Saturation.

The optimal MI algorithm was rf, which was selected based on the degree of overlap between the true values and the imputed values (Supplemental Digital Content, Fig. S2, http://links.lww.com/JS9/E461). This process was repeated 50 times, and the mean value was taken to impute the missing data for further analysis.

### Algorithm and variables analysis

**Figure 2.**
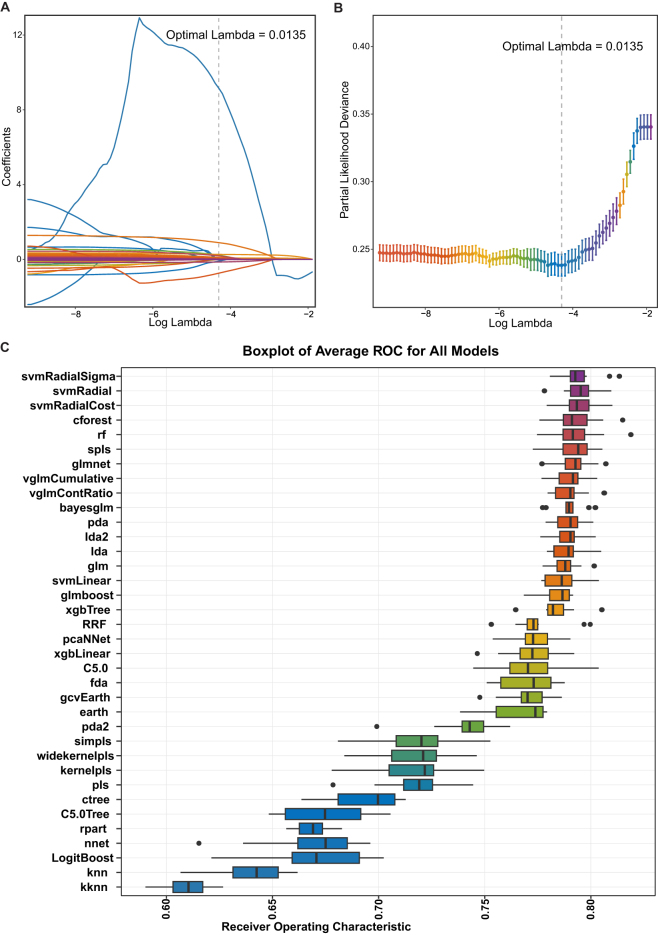
Identification and screening of model variables and algorithms for predicting 26-day mortality. (A) The lasso coefficient analysis. *Log(lambda)*: Log Regularization Parameter, X axis represented the log of the regularization parameter lambda. *Coefficients*: Y axis showed the feature coefficients of the variables in the model. These coefficients reflected the degree to which each feature affects the model’s predictions. Each line represented a different variable, and the value of the feature coefficient for each variable is plotted against the Log(lambda). The dotted line represented the corresponding feature coefficient of each variable under the optimal lambda value. (B) The relationship between partial likelihood deviance and the regularization parameter lambda in the lasso model. *Log(lambda)*: Log Regularization Parameter, X axis represented the log of the regularization parameter *lambda. Partial Likelihood Deviance*: Y axis showed the partial likelihood deviance for different values of the regularization parameter. Partial likelihood deviance quantifies model fit (lower values indicate better predictive performance). Dots represented the average partial likelihood deviance with the standard deviation of the partial likelihood deviance. The dotted line indicated the minimum lambda by ten-fold cross-validation. (C) Average AUC values for multiple ML algorithms. *AUC* area under the curve.

Initially, Lasso regression analysis was performed to refine the selection of predictive variables. First, Lasso coefficient analysis was conducted on the variables (Fig. 2A), followed by ten-fold cross-validation to determine the optimal lambda value (lambda.min = 0.0135). This resulted in a model that included 32 variables and demonstrated superior performance (Fig. 2B). Additionally, the kappa value (also known as the condition number, reflecting multicollinearity [visualized in Supplemental Digital Content, Fig. S3 http://links.lww.com/JS9/E461]) of the design matrix decreased from 985.0592 to 59.1384 after Lasso analysis, indicating a significant reduction in multicollinearity among the selected variables. The mean AUC for each algorithm model was evaluated using ten-fold cross-validation (Fig. 2C).

**Figure 3.**
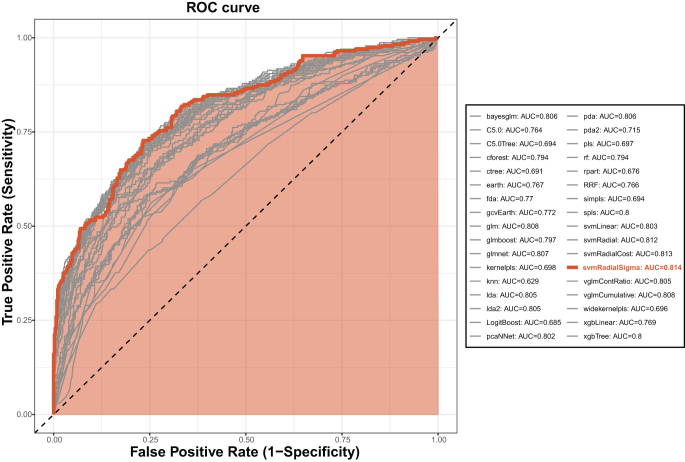
ROC curves of each ML algorithm for predicting 26-day mortality using trial set. *ROC* Receiver Operating Characteristic, *AUC* Area Under the Curve. The red curve highlighted the best ML algorithms, and the rest of the algorithms were represented by gray curves.

**Figure 4.**
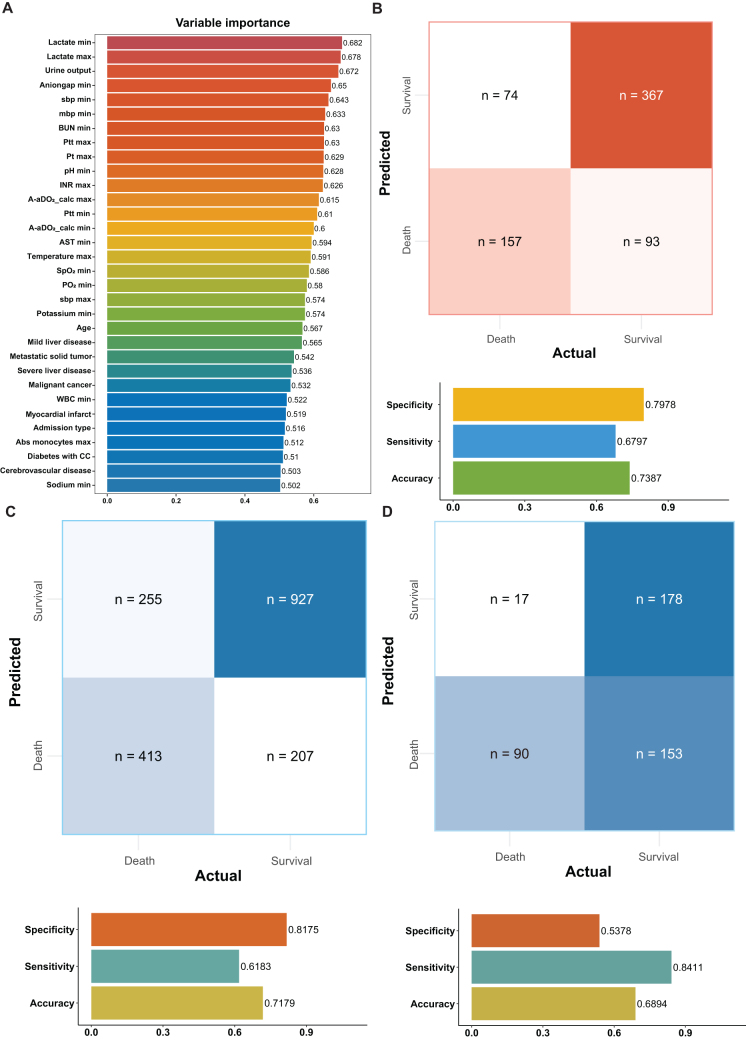
Variable importance analysis and discrimination verification of SAFE-Mo. (A) Visualization of variable importance. (B) Confusion matrix and discrimination metrics in internal validation (C) Confusion matrix and discrimination metrics in external validation set one. (D) Confusion matrix and discrimination metrics in external validation set two.

The AUC of ML algorithm models were calculated using the training set, alongside accuracy, precision, recall, F1-score, specificity, sensitivity, NPV, and PPV for comprehensive model diagnostics. Based on these evaluations, the SVM with radial basis function kernel and sigma optimization (svmRadialSigma) was selected as the optimal algorithm, designated as the only algorithm of SAFE-Mo (Supplemental Digital Content, Table 2, Table S5 http://links.lww.com/JS9/E466, and Figure 3).Variable importance analysis within SAFE-Mo identified the ranking of variables. Variables with an importance score exceeding 65 included lactate max, lactate min, urine output, and anion gap min (Fig. 4A).

**Table 2:** Evaluation of each ML algorithm

Method	Accuracy	Precision	Recall	F1-score	Specificity	Sensitivity	NPV	PPV
svmRadialSigma	0.75 (0.72-0.78)	0.61 (0.51-0.68)	0.72 (0.64-0.86)	0.66 (0.62-0.71)	0.77 (0.64-0.83)	0.72 (0.64-0.86)	0.85 (0.81-0.91)	0.61 (0.51-0.68)
glm	0.74 (0.71-0.78)	0.60 (0.51-0.73)	0.71 (0.57-0.87)	0.65 (0.60-0.70)	0.76 (0.61-0.89)	0.71 (0.57-0.87)	0.84 (0.80-0.90)	0.60 (0.51-0.73)
glmnet	0.74 (0.71-0.78)	0.60 (0.54-0.69)	0.72 (0.63-0.81)	0.66 (0.61-0.70)	0.76 (0.67-0.85)	0.72 (0.63-0.81)	0.85 (0.81-0.89)	0.60 (0.54-0.69)
lda	0.74 (0.71-0.78)	0.60 (0.52-0.71)	0.73 (0.61-0.82)	0.66 (0.60-0.71)	0.75 (0.65-0.86)	0.73 (0.61-0.82)	0.85 (0.81-0.89)	0.60 (0.52-0.71)
pcaNNet	0.74 (0.71-0.78)	0.60 (0.54-0.67)	0.73 (0.65-0.79)	0.66 (0.61-0.70)	0.75 (0.72-0.83)	0.73 (0.65-0.79)	0.85 (0.81-0.89)	0.60 (0.54-0.67)
spls	0.74 (0.70-0.78)	0.63 (0.52-0.70)	0.68 (0.58-0.83)	0.65 (0.60-0.70)	0.80 (0.63-0.86)	0.68 (0.58-0.83)	0.83 (0.80-0.88)	0.63 (0.52-0.70)
svmLinear	0.74 (0.71-0.78)	0.58 (0.51-0.72)	0.74 (0.57-0.85)	0.65 (0.62-0.70)	0.73 (0.65-0.90)	0.74 (0.57-0.85)	0.85 (0.81-0.90)	0.58 (0.51-0.72)
svmRadial	0.74 (0.71-0.78)	0.59 (0.53-0.68)	0.73 (0.65-0.82)	0.65 (0.61-0.71)	0.75 (0.66-0.86)	0.73 (0.65-0.82)	0.85 (0.81-0.90)	0.59 (0.53-0.68)
svmRadialCost	0.74 (0.72-0.78)	0.58 (0.52-0.71)	0.76 (0.62-0.84)	0.66 (0.62-0.71)	0.72 (0.65-0.87)	0.76 (0.62-0.84)	0.86 (0.81-0.90)	0.58 (0.52-0.71)
vglmContRatio	0.74 (0.70-0.78)	0.61 (0.51-0.71)	0.72 (0.61-0.83)	0.66 (0.60-0.70)	0.77 (0.64-0.87)	0.72 (0.61-0.83)	0.84 (0.81-0.89)	0.61 (0.51-0.71)
vglmCumulative	0.74 (0.71-0.78)	0.63 (0.50-0.73)	0.66 (0.59-0.86)	0.65 (0.61-0.70)	0.81 (0.62-0.88)	0.66 (0.59-0.86)	0.83 (0.80-0.91)	0.63 (0.50-0.73)
xgbTree	0.74 (0.71-0.78)	0.61 (0.50-0.73)	0.70 (0.58-0.84)	0.65 (0.61-0.70)	0.78 (0.61-0.89)	0.70 (0.58-0.84)	0.84 (0.80-0.88)	0.61 (0.50-0.73)
bayesglm	0.73 (0.71-0.77)	0.60 (0.50-0.73)	0.69 (0.59-0.84)	0.65 (0.60-0.70)	0.77 (0.62-0.89)	0.69 (0.59-0.84)	0.83 (0.80-0.90)	0.60 (0.50-0.73)
glmboost	0.73 (0.70-0.77)	0.64 (0.53-0.71)	0.65 (0.60-0.81)	0.65 (0.60-0.69)	0.82 (0.64-0.87)	0.65 (0.60-0.81)	0.82 (0.80-0.88)	0.64 (0.53-0.71)
lda2	0.73 (0.71-0.78)	0.65 (0.50-0.70)	0.65 (0.59-0.84)	0.65 (0.60-0.70)	0.82 (0.63-0.85)	0.65 (0.59-0.84)	0.82 (0.81-0.89)	0.65 (0.50-0.70)
pda	0.73 (0.70-0.77)	0.57 (0.52-0.75)	0.74 (0.57-0.82)	0.65 (0.60-0.70)	0.72 (0.64-0.90)	0.74 (0.57-0.82)	0.85 (0.80-0.88)	0.57 (0.52-0.75)
rf	0.73 (0.70-0.77)	0.65 (0.54-0.78)	0.63 (0.56-0.77)	0.64 (0.60-0.69)	0.83 (0.70-0.92)	0.63 (0.56-0.77)	0.82 (0.79-0.87)	0.65 (0.54-0.78)
cforest	0.72 (0.69-0.76)	0.66 (0.49-0.77)	0.60 (0.50-0.85)	0.63 (0.57-0.68)	0.85 (0.62-0.92)	0.60 (0.50-0.85)	0.81 (0.77-0.89)	0.66 (0.49-0.77)
RRF	0.71 (0.68-0.74)	0.60 (0.52-0.69)	0.63 (0.54-0.73)	0.62 (0.57-0.66)	0.79 (0.67-0.86)	0.63 (0.54-0.73)	0.81 (0.77-0.85)	0.60 (0.52-0.69)
fda	0.71 (0.68-0.74)	0.64 (0.50-0.71)	0.58 (0.51-0.77)	0.61 (0.55-0.66)	0.84 (0.64-0.88)	0.58 (0.51-0.77)	0.80 (0.77-0.85)	0.64 (0.50-0.71)
xgbLinear	0.71 (0.67-0.75)	0.64 (0.48-0.71)	0.57 (0.53-0.82)	0.60 (0.56-0.67)	0.84 (0.58-0.88)	0.57 (0.53-0.82)	0.80 (0.77-0.88)	0.64 (0.48-0.71)
C5.0	0.70 (0.67-0.74)	0.55 (0.49-0.75)	0.68 (0.47-0.81)	0.61 (0.55-0.66)	0.72 (0.60-0.92)	0.68 (0.47-0.81)	0.82 (0.76-0.87)	0.55 (0.49-0.75)
earth	0.70 (0.67-0.74)	0.56 (0.48-0.70)	0.65 (0.53-0.78)	0.60 (0.56-0.66)	0.75 (0.61-0.87)	0.65 (0.53-0.78)	0.81 (0.77-0.86)	0.56 (0.48-0.70)
gcvEarth	0.70 (0.68-0.74)	0.57 (0.46-0.66)	0.65 (0.58-0.82)	0.61 (0.58-0.67)	0.76 (0.59-0.84)	0.65 (0.58-0.82)	0.81 (0.78-0.88)	0.57 (0.46-0.66)
pda2	0.68 (0.65-0.73)	0.59 (0.51-0.70)	0.56 (0.45-0.63)	0.58 (0.51-0.63)	0.80 (0.73-0.88)	0.56 (0.45-0.63)	0.79 (0.75-0.83)	0.59 (0.51-0.70)
kernelpls	0.67 (0.64-0.72)	0.55 (0.47-0.63)	0.57 (0.48-0.73)	0.56 (0.51-0.63)	0.77 (0.61-0.83)	0.57 (0.48-0.73)	0.78 (0.75-0.83)	0.55 (0.47-0.63)
pls	0.67 (0.64-0.72)	0.55 (0.47-0.65)	0.58 (0.47-0.73)	0.56 (0.51-0.63)	0.76 (0.60-0.85)	0.58 (0.47-0.73)	0.78 (0.75-0.83)	0.55 (0.47-0.65)
simpls	0.67 (0.64-0.71)	0.54 (0.46-0.64)	0.58 (0.44-0.75)	0.56 (0.50-0.63)	0.75 (0.58-0.87)	0.58 (0.44-0.75)	0.78 (0.75-0.83)	0.54 (0.46-0.64)
widekernelpls	0.67 (0.64-0.72)	0.54 (0.47-0.63)	0.58 (0.48-0.73)	0.56 (0.50-0.63)	0.75 (0.59-0.83)	0.58 (0.48-0.73)	0.78 (0.75-0.83)	0.54 (0.47-0.63)
C5.0Tree	0.65 (0.61-0.70)	0.56 (0.40-0.61)	0.49 (0.41-0.88)	0.52 (0.46-0.60)	0.80 (0.40-0.85)	0.49 (0.41-0.88)	0.76 (0.72-0.88)	0.56 (0.40-0.61)
rpart	0.64 (0.60-0.67)	0.60 (0.54-0.74)	0.43 (0.30-0.46)	0.50 (0.41-0.54)	0.86 (0.83-0.93)	0.43 (0.30-0.46)	0.75 (0.71-0.78)	0.60 (0.54-0.74)
ctree	0.63 (0.60-0.69)	0.48 (0.43-0.55)	0.58 (0.52-0.75)	0.53 (0.49-0.60)	0.69 (0.56-0.76)	0.58 (0.52-0.75)	0.77 (0.74-0.83)	0.48 (0.43-0.55)
nnet	0.63 (0.59-0.66)	0.56 (0.49-0.72)	0.42 (0.28-0.52)	0.48 (0.37-0.55)	0.83 (0.77-0.93)	0.42 (0.28-0.52)	0.74 (0.70-0.78)	0.56 (0.49-0.72)
knn	0.59 (0.56-0.63)	0.52 (0.38-0.65)	0.33 (0.18-0.69)	0.41 (0.28-0.54)	0.84 (0.48-0.94)	0.33 (0.18-0.69)	0.72 (0.68-0.77)	0.52 (0.38-0.65)
LogitBoost	0.58 (0.55-0.60)	0.75 (0.61-0.86)	0.18 (0.13-0.24)	0.29 (0.22-0.37)	0.97 (0.95-0.98)	0.18 (0.13-0.24)	0.70 (0.67-0.75)	0.75 (0.61-0.86)
kknn	0.50 (0.50-0.50)	Na	0.00 (0.00-0.00)	Na	1.00 (1.00-1.00)	0.00 (0.00-0.00)	0.67 (0.67-0.67)	Na

Accuracy: (TP + TN)/(P + N)—Overall classification correctness

Precision (PPV): TP/(TP + FP)—Positive predictive value

Recall (Sensitivity): TP/(TP + FN)—True positive rate

Specificity: TN/(TN + FP)—True negative rate

F1-score: 2*(Precision*Recall)/(Precision + Recall)—Harmonic mean

NPV: TN/(TN + FN)—Negative predictive value

All performance metrics were computed with 95% confidence intervals derived through non-parametric bootstrap resampling.

N, negative; NPV, negative predictive value; P, positive; PPV, positive predictive value; TN, true negative; TP, true positive.

### Model validation

The performance of SAFE-Mo was validated using an internal validation set. In the confusion matrix, the number of 26-day mortality true negative results was 367, true positive results were 157, false negatives numbered 74, and false positives were 93. SAFE-Mo achieved a specificity/sensitivity/accuracy of 0.7978, 0.6797, and 0.7387, respectively (Fig. 4B). Further validation of SAFE-Mo’s performance was conducted using external validation sets one and two. In the set one, the confusion matrix showed 927 true negative results, 413 true positive results, 207 false negatives, and 255 false positives based on 26-day mortality. The specificity, sensitivity, and accuracy for SAFE-Mo were 0.8175, 0.6183, and 0.7179, respectively (Fig. 4C). In external validation set two, the confusion matrix demonstrated 178 true negative results, 90 true positive results, 153 false negatives, and 17 false positives based on 26-day mortality. The specificity, sensitivity, and accuracy for SAFE-Mo were 0.5378, 0.8411, and 0.6894, respectively (Fig. 4D)

### Model comparison

**Figure 5.**
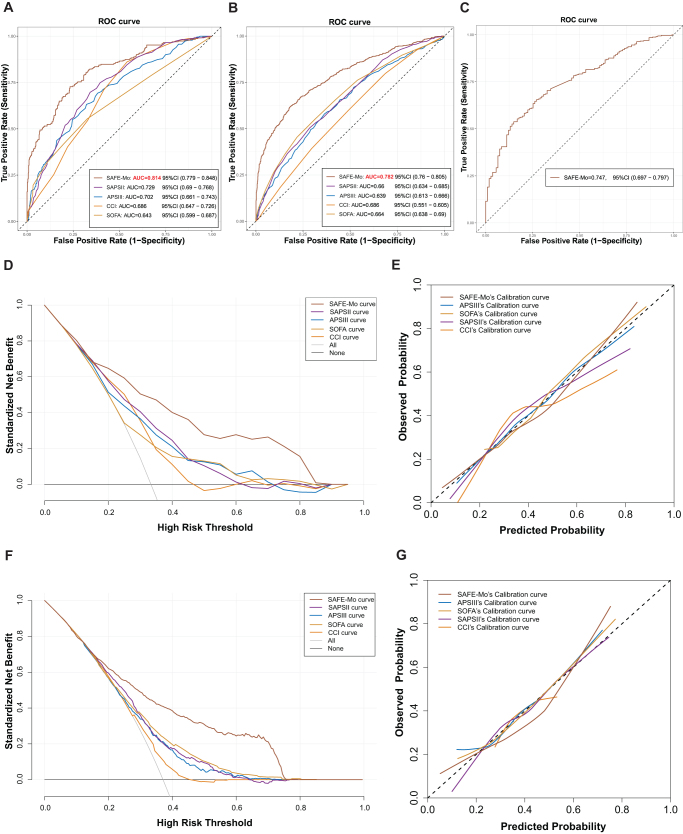
ROC curves and the clinical utility assessment of SAFE-Mo, APSIII, SAPS II, SOFA, and CCI for predicting 26-day mortality using validation set. (A) ROC curves in internal validation. (B) ROC curves in external validation set one. (C) SAFE-Mo’ ROC curve in external validation set two. (D) DCA of each model using the internal validation set. (E) Calibration curves of each model in internal validation (F) DCA of each model using external validation set one (G) Calibration curves of each model in external validation set one. *DCA* decision curve analysis; *SAFE-Mo* Sepsis-associated ARDS fatality evaluation model.

Comparative performance evaluation of SAFE-Mo against four clinical risk assessment models was conducted using the internal validation set. Figure 5A demonstrates superior discriminative capacity of SAFE-Mo in 26-day mortality prediction (AUC = 0.814). Univariate logistic regression analyses revealed statistically significant odds ratio (OR) variations across all models, with SAFE-Mo exhibiting the maximal effect size (OR = 168.94, 95% CI 74.91–381.01; *P*<0.001). Multivariate regression models confirmed significant predictive associations for SAFE-Mo (adjusted OR = 55.17, 95% CI 20.20–150.71), CCI, and SOFA scores, where SAFE-Mo maintained the strongest prognostic relationship (Supplemental Digital Content, Table S4 http://links.lww.com/JS9/E465; Fig. S4A http://links.lww.com/JS9/E461). Correlation analyses identified the highest association between SAFE-Mo and SAPSII (Pearson’s *r* = 0.63, *P*<0.001) (Supplemental Digital Content, Fig. S4B http://links.lww.com/JS9/E461).

The analysis of external validation set one replicated these findings, with SAFE-Mo achieving the predictive accuracy (AUC = 0.782) for 26-day mortality (Fig. 5B). Univariate comparisons demonstrated significant OR heterogeneity among models, where SAFE-Mo retained the most substantial risk association (OR = 80.59, 95% CI 50.03–129.82). Multivariate adjustments preserved significant predictive values for SAFE-Mo (OR = 38.86, 95% CI 23.07–65.47), SAPSII, and SOFA scores, confirming SAFE-Mo’s persistent dominance (Supplemental Digital Content, Table S5, http://links.lww.com/JS9/E466; Fig. S4C, http://links.lww.com/JS9/E461). Notably, correlation patterns shifted in this cohort, showing maximal association between SAFE-Mo and SOFA score (*r* = 0.42, *P*<0.001) (Supplemental Digital Content, Fig. S4D, http://links.lww.com/JS9/E461). Analyses utilizing external validation set two evaluated the model’s clinical adaptability, demonstrating preserved 26-day mortality prediction accuracy (AUC = 0.747) under four-variable missing conditions (Fig. 5C).

### Clinical utility

To objectively assess the clinical utility of SAFE-Mo in predicting 26-day mortality, DCA and calibration analysis were conducted. In the internal validation set, DCA results indicated that SAFE-Mo had an appropriate risk threshold range of 0.2 to 0.8. Within this range, net clinical benefit was higher (Fig. 5D). Calibration curve analysis demonstrated consistency between predicted probabilities and actual outcomes for APS III, SOFA, and SAFE-Mo; the predicted probabilities from SAFE-Mo were slightly overestimated compared to the actual outcomes (Fig. 5E).

In the external validation set, overall, SAFE-Mo had an appropriate risk threshold range of 0.2 to 0.7, indicating higher net clinical benefit within this range (Fig. 5F). Calibration curve analysis showed that APS III, SAPS II, and SOFA maintained a high level of consistency between predicted and actual outcomes. Notably, when actual probabilities were between 0.2 and 0.58, the predicted probabilities from SAFE-Mo were overestimated compared to the actual outcomes (Fig. 5G). Considering the potential possibility of our model being used in clinical practice in the future, a simple front-end web page was made (Supplemental Digital Content, Table S6, http://links.lww.com/JS9/E467) to facilitate clinicians to quickly view the patients’ SAFE-Mo index and preliminarily determine their mortality risk and provide better clinical resource utilization.

## Discussion

Mortality rates in patients with sepsis-associated ARDS remain high, with only minimal improvements observed in recent years, now stabilizing at approximately 40%^[1,7,26]^. Therefore, constructing a predictive model capable of early identification of patients likely to experience mortality is essential for guiding appropriate interventions and optimizing resource allocation. This study developed and validated a clinical prediction model, SAFE-Mo, using three independent databases: the MIMIC-IV, eICU CRD, and NWICU. SAFE-Mo demonstrated good calibration and discrimination in internal validation and external validation set one. In clinical practice, the lack of partial information about patients is a common situation. In the external validation set two where four pieces of information were missing, the AUC of SAFE-Mo was still 0.747, which indicated that SAFE-Mo had sufficiently stable predictive power. Predictors included in SAFE-Mo are biologically plausible, encompassing demographic data, vital signs, serological markers, and comorbidity indicators, all readily extractable from medical records. SAFE-Mo performed excellently in predicting early mortality risk in patients with sepsis-associated ARDS, providing strong support for clinical decision-making.

Parameters within the model exhibit multicollinearity, which can exacerbate the tension between optimization and generalization, ultimately leading to model overfitting and hindering the generalizability of discriminant functions^[27]^. To enhance the reliability of selected variables, Lasso regression analysis was used to address collinearity issues. After processing, kappa values indicated a significant reduction in collinearity. A total of 32 variables were included in SAFE-Mo. Post-modeling variable importance analysis revealed that lactate min, lactate max, urine output, and anion gap min had high explanatory power.

Lactate emerged as the most critical variable in SAFE-Mo, with significant predictive and prognostic value. In critically ill patients with sepsis, lactate levels frequently increase, reflecting a mismatch between production and clearance rates. Patients with lung injury produce lactate more rapidly^[28]^, possibly due to inflammatory cytokines released by immune cells and elevated catecholamine levels post-stress, promoting glycolysis. Additionally, lactate clearance significantly decreases in sepsis patients^[29–32]^. Given that lactate is influenced by multiple factors, relying solely on lactate levels to determine tissue hypoxia is not advisable^[33]^. The anion gap serves as an important indicator of acid-base balance^[34]^. Rapid increases in lactate levels in sepsis-related ARDS patients can easily lead to acid-base disturbances, elevating the anion gap. The lungs, as one of the target organs for sepsis and an acid-base regulatory organ, can cause rapid systemic acid-base imbalances when dysfunctional. Key variables in SAFE-Mo include lactate, anion gap, sodium, potassium, and pH, all reflecting the body’s acid-base status. This suggests that acid-base balance may be a critical factor influencing early mortality risk in sepsis-related ARDS patients.

Urine output, another key variable, reflects circulatory volume and fluid management. Many studies indicate that low urine output correlates with higher mortality in sepsis patients^[35,36]^. However, further research is needed when the urine output/fluid intake ratio is ≥0.5^[37]^. Therefore, dynamic fluid management is necessary for sepsis-related ARDS patients rather than absolute urine output values.

Combining baseline data with variable importance analysis revealed that comorbidities involving organs, particularly the heart, liver, and kidneys, substantially influenced early mortality risk in patients. The crosstalk between these organs constitutes one of the main mechanisms by which the body performs normal physiological functions and sustains life^[38]^. Patients with sepsis are more susceptible to organ dysfunction, and the presence of comorbidities affecting multiple organs further disrupts inter-organ communication. Consequently, imbalances in respiration, blood pressure, organ perfusion, and coagulation contribute to high mortality risks. Malignant cancers and metastatic tumors also emerged as critical factors, as they or the immunotherapy received by patients can impact both innate and adaptive immunity; they may exacerbate the severity of sepsis and complicate clinicians’ assessments of patient immune status^[39]^. Therefore, focusing on the management of comorbidities and implementing personalized precision therapies can help mitigate the early mortality risk in patients with sepsis-associated ARDS.

Given the time of deaths in the training set is skewed, the median is statistically a better way to describe the overall average. Median survival time is used to differentiate early from late mortality. In this study, early mortality was defined as occurring within 26 days, aiding in identifying patients who may respond poorly to treatment or have more severe conditions^[40]^. SAFE-Mo was compared against various ML algorithms and traditional clinical risk prediction models (including SOFA, APS III, SAPSII, and CCI). Both internal and external validations confirmed SAFE-Mo’s superior AUC. Further DCA curve analyses validated SAFE-Mo’s clinical utility, demonstrating the largest reasonable risk threshold probability range and highest net benefit compared to other models. We also compared SAFE-Mo with a recently published Clinical Prediction Model (CPM) (Supplemental Digital Content, Table S7 http://links.lww.com/JS9/E763). In predicting early mortality risk, SAFE-Mo demonstrated superior performance relative to the CPM. Calibration curves indicated that SAFE-Mo slightly overestimated mortality risk overall. Several factors may underlie this result, the most plausible being a mismatch in baseline risk between the training and validation datasets. In the development cohort, the event rate may have been higher than in our validation cohort, causing the model to overestimate risk among individuals who are in fact at low risk. Alternatively, a limited sample size and a small number of events can make the model prone to overfitting the high-risk cases in the training data when positive outcomes are scarce. Clinically, this tendency of SAFE-Mo may be advantageous: by amplifying early risk signals, it can increase the sensitivity of warnings, reduce missed detections of rare but critical events, and thereby maximize the benefits of prompt intervention. Although SAFE-Mo exhibits a slight overall overestimation of risk at the calibration level, decision-analytic and cost-effectiveness considerations suggest that this bias may improve early warning performance, enhance patient safety, and provide strong impetus for subsequent model recalibration and ongoing learning.

Overall, compared to traditional models, SAFE-Mo exhibited superior predictive capability of early mortality. SAFE-Mo has the potential to assist clinicians in identifying high-risk patients early, like patients with unusually high levels of lactate in sepsis-associated ARDS, assessing prognosis, and facilitating risk-adjusted comparisons of center-specific outcomes. Practical advantages in clinical settings include guiding personalized treatment strategies, determining the need for aggressive interventions, and optimizing resource utilization. SAFE-Mo also aids in deciding the most appropriate care environment, such as distinguishing patients requiring transfer to the ICU for close monitoring and intensive therapy from those who can receive routine care in non-ICU settings.

In our study, several limitations should be acknowledged. First, the retrospective and observational nature of the study may introduce selection bias. Treatment and medication protocols evolved over time during the data collection phase, potentially affecting patient outcomes and mortality risks. Second, while the MIMIC-IV database originates from a single United States center, the use of the multi-center eICU CRD database for validation might limit SAFE-Mo’s applicability to sepsis-associated ARDS patients in other countries/regions. In the NWICU dataset, the median from MIMIC-IV is used to replace the very few 100% missing variables, which is a necessary trade-off in the real world, but it may affect the independence of external validation to a certain extent, and a more complete validation set data should be obtained to supplement it in the future ideal scenario. Absence of multicenter prospective cohort studies hinders broader generalization. Lastly, despite imputed data being similar to true results, some selection bias remains possible. Moreover, imaging data and other outcome-influencing factors such as treatment strategies and nursing levels were not considered. Thereby, further validation in prospective, multicenter, large-scale patient datasets will provide stronger evidence for the efficacy and generalizability of SAFE-Mo.

## Conclusion

This study utilized the MIMIC-IV, eICU CRD, and NWICU databases to construct and validate a ML model, SAFE-Mo, which predicts early mortality in patients with sepsis-associated ARDS and outperforms traditional prediction models across all metrics. SAFE-Mo can guide clinicians to focus on critical indicators such as lactate, urine output, anion gap, and others, enabling appropriate measures to improve clinical outcomes for high-risk patients.

## Data Availability

The data that support the findings of this study are available from PhysioNet (https://physionet.org/) but restrictions apply to the availability of these data, which were used under license for the current study, and so are not publicly available. Data are, however, available from the authors upon reasonable request and with permission of PhysioNet (https://physionet.org/). The complete analysis code, including data preprocessing, model development, and validation pipelines, has been deposited in our GitHub repository: https://github.com/ChutingY/SAFE-Mo.
